# Nasofrontal surgical reconstruction by external table flap of frontal bone following removal of a dermoid cyst revealed by a fistula: A case report and review of the literature

**DOI:** 10.3205/iprs000127

**Published:** 2019-01-30

**Authors:** Kamal Chtira, Yassine Elallouchi, Farid Zahrou, Mouhssine Assamadi, Abdelaziz Ait El Qadi, Houssaine Ghannane, Mehdi Laghmari

**Affiliations:** 1 Neurosurgery Department, Ibn Tofail Hospital, University Hospital Mohammed VI, Marrakesh, Morocco; 2 Neurosurgery Department, Arrazi Hospital, University Hospital Mohammed VI, Marrakesh, Morocco

**Keywords:** nasofrontal fistula, dermoid cyst, surgical treatment, nasofrontal reconstruction

## Abstract

Nasofrontal fistulas correspond to the persistence of an abnormal communication of embryological origin between the deep layer of the skin and the central nervous system (CNS). They can rarely be associated with a dermoid cyst and be revealed by a locoregional infection, and especially neuromeningeal infections can be serious. The treatment is mainly surgical by performing a total excision of the cyst and the repair of defects. The authors report the case of an 18-month-old infant operated for a dermoid cyst revealed by a nasofrontal fistula. They insist on the characteristics of this pathology in order to establish a diagnosis and an early treatment to avoid the complications that can be heavy in certain cases. They describe the steps of nasofrontal reconstruction by a small flap taken from the outer table of the frontal bone with better esthetic results.

## Introduction

The nasofrontal dermoid cyst is a rare abnormality of embryological development and may be revealed by a nasofrontal fistula that corresponds to the absence or incomplete obliteration of the neuroectoderm during the development of the frontonasal region. The progressive enlargement of the dermoid cyst can lead to skeletal deformation, soft tissue, and a locoregional infection, especially a neuromeningeal infection, which can be serious. Early diagnosis and appropriate treatment are important to avoid neuromeningeal and esthetic complications [1], [2]. We report the medical observation of an infant operated for a dermoid cyst revealed by a nasofrontal fistula, highlight its semiological, radiological, and therapeutic characteristics for early management, and describe the steps of this minimally invasive surgical reconstruction.

## Case description

**Figure 1:**
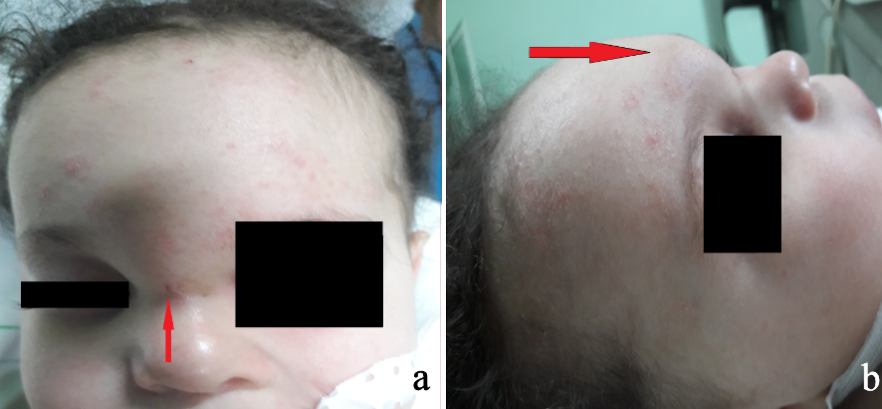
Frontal (a) and profile (b) images of the patient. Facial image of the face (a) showing the mass and the fistula’s orifice indicated by the arrow and of the profile of the face (b) showing the frontal mass in the shape of a small arch facing the glabella

**Figure 2:**
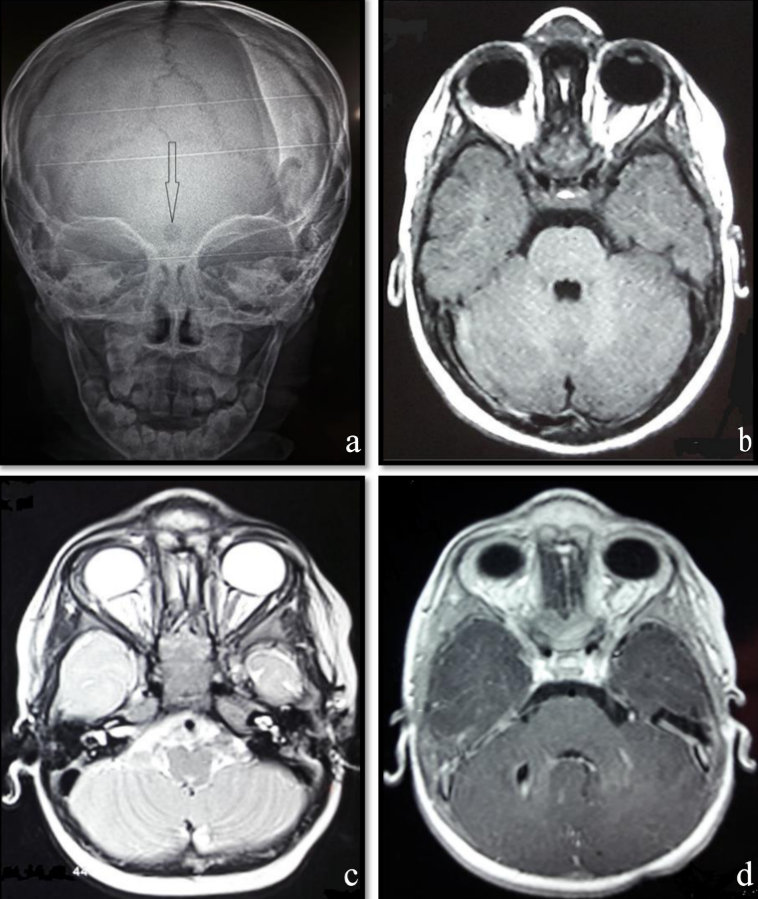
Frontal radiograph of the skull (a) showing nasofrontal bone defect and axial brain MRI weighted sequences T1 (b), T2 (c), and T1 contrast enhanced (d). The lesion sits in the medial basifrontal and appears hypointense on T1 and hyperintense on T2 sequences, and enhances after injection of the contrast product (d).

**Figure 3:**
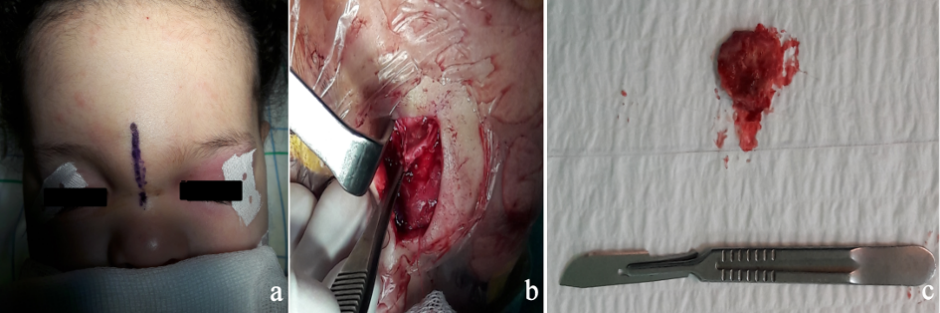
Preoperative image (a) showing the marking of the incision, intraoperative image (b) made during the excision of the cyst, and image of the cyst after complete excision (c)

**Figure 4:**
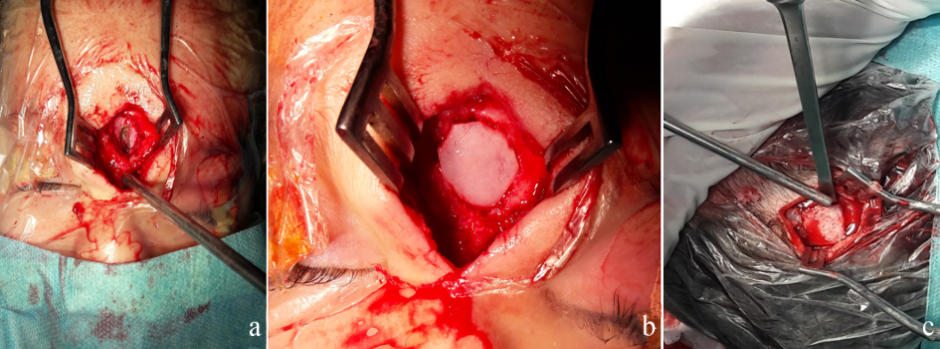
Intraoperative image showing the defect of the bone and the dura after removal of the cyst (a). The clogging of the bone defect by the external table of the frontal bone (b), then an intraoperative view when taking a small flap of the external table of the frontal bone (c).

**Figure 5:**
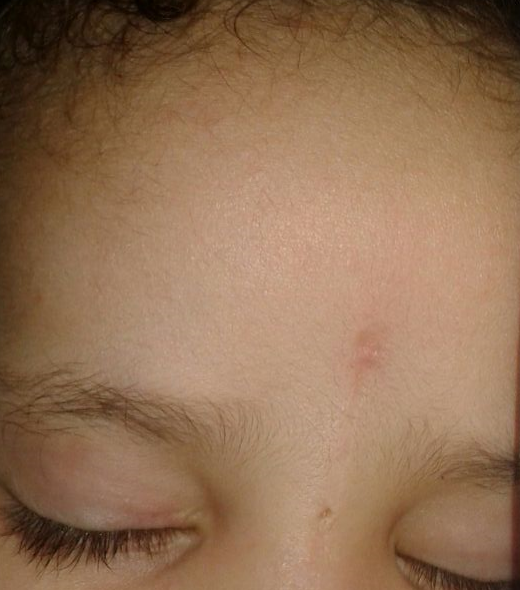
Image showing the esthetic result obtained at 10 months after surgery

An 18-month-old infant without specific pathological history presented at 1 month of admission a median frontal swelling facing the nasion associated with a citrin yellow flow of increasing intensity without fever. Clinical examination showed a nasofrontal fistula allowing a flow to the top of a small inflammatory mass slightly sensitive to palpation (Figure 1 (Fig. 1)) evolving in a context of apyrexia and conservation of the general state. The frontal standard radiography of the skull showed a medial frontal bone defect (Figure 2a (Fig. 2)). Magnetic resonance imaging (MRI) of the brain (Figure 2b–d (Fig. 2)) demonstrated a median basifrontal cystic lesion in favor of a fistula associated with a nasofrontal cyst, which appears hypointense on T1 (Figure 2b (Fig. 2)) and hyperintense on T2 (Figure 2c (Fig. 2)) and enhances after gadolinium injection (Figure 2d (Fig. 2)), underlining that the infectious assessment was normal. The patient was operated by performing a small, median nasofrontal incision centered on the mass (Figure 3a (Fig. 3)), careful dissection around the cyst (Figure 3b (Fig. 3)), and its complete removal (Figure 3c (Fig. 3)). The defect of the dura mater (Figure 4a (Fig. 4)) was closed by making hermetic sutures, and the bone defect was clogged by a small flap of the external table taken from the frontal bone in precoronal (Figure 4a–b (Fig. 4)). The incision was esthetically closed by continuous absorbable intradermal sutures. The postoperative follow-up was simple without any incidents with excellent healing of the wound. Histological examination confirmed the diagnosis of the dermoid cyst and we report a very satisfying aesthetic result obtained 10 months after the surgical intervention (Figure 5 (Fig. 5)).

## Discussion

During the development of the skull base and the nose, the mesenchymal structures are formed by the fusion of several structures that ossify. But before fusion, there are recognized spaces between these structures that include the fonticulus frontalis, the prenasal space, and the foramen caecum, which are normally closed by fusion and ossification [3], [4]. During the bone closure of fonticulus frontalis with foramen caecum, the dural diverticulum travels through the prenasal space and comes into contact with the nasal skin, at the time the frontal bone develops, the skin and dura separate, and the projection of the dura mater is encircled by the foramen caecum [4]. The failure of the obliteration of these spaces and the persistence of variable portions of this diverticulum lead to the group of median nasofrontal masses of which the dermoid cyst is a part [5]. The dermoid cyst is a benign tumor and contains adnexal structures, such as skin, hair follicles, and sebaceous glands [5]. The nasofrontal locations of neuro-cutaneous fistulas are exceptional, less than 5%. They are asymptomatic and their discovery is fortuitous in 58% of cases [6]. In one third of cases, they are revealed by a locoregional infection or by a painless swelling of the nasal dorsum, which is generally mobile with respect to the superficial plane and fixed with respect to the deep planes [1]. The presence of a fistulous opening on the nasal surface is pathognomonic. Patients may also have glabella edema, nasal enlargement, or hypertelorism. Intracranial complications are severe and revealing in about 30% of cases. It may be meningitis, brain abscess, frontonasal osteomyelitis, and seizures [7]. MRI is useful for exploring the cyst and its intracranial and nasal extension, as well as its relationship with neighboring structures [7]. The intracranial portion of the lesion remains most often extra-axial and may involve the dura. An intra-parenchymal cerebral extension is rare. CT scan is used to identify the site of the bone defect in case of an intracranial extension and to reveal lesions of neighboring structures [1]. Complete resection of the cyst and the nasofrontal fistula remains the effective and definitive treatment. However, thorough preoperative evaluation is essential before excision of the nasofrontal dermoid cyst.

Different approaches are described in the literature ranging from a simple incision with cyst ablation to lateral and transverse rhinotomy. A bicoronal approach in case of an intracranial extension of the cyst is sometimes necessary [8], [9]. Any bone defects in the skull must be repaired. Currently, and for intracranial extensions, minimal invasive approaches are recommended [10]. A frontal incision, with the creation of a small bony flap facing the cyst, allows its removal with less morbidity than the bicoronal approach.

Finally, it is important to insist on the complete excision of the cyst. Indeed, a partial resection exposes to recurrence in 50 to 70% [11]. Fistulas and nasofrontal cysts have an excellent prognosis if they are well managed, except in cases of of severe neuromeningeal infections.

## Conclusion

The knowledge of the embryological development of the nose and the skull base is important because a defect of obliteration and embryological communication between the nasal region and the dura can lead to a nasofrontal fistula, which must be excluded as a diagnose in cases of a nasofrontal discharge especially in infants and small children, and may reveal a dermoid cyst. Imaging helps in diagnosis and choice of treatment. A complete surgical excision is essential as well as bone and dura defect repair to avoid infection.

## Notes

### Competing interests

The authors declare that they have no competing interests.
